# Exploring the significance of interleukin-33/ST2 axis in minimal change disease

**DOI:** 10.1038/s41598-023-45678-z

**Published:** 2023-10-31

**Authors:** Nobuhiro Kanazawa, Masayuki Iyoda, Taihei Suzuki, Shohei Tachibana, Ryuichi Nagashima, Hirokazu Honda

**Affiliations:** 1 Division of Nephrology, Department of Medicine, Showa University School of Medicine, Tokyo, Japan; 2 Department of Microbiology and Immunology, Showa University School of Medicine, 1-5-8 Hatanodai, Shinagawa-ku, Tokyo, 142-8555 Japan

**Keywords:** Immunology, Nephrology

## Abstract

Minimal change disease (MCD), a common cause of idiopathic nephrotic syndrome, has been postulated to exhibit an association with allergic conditions. Recent studies revealed the crucial role of interleukin (IL)-33 in type 2 innate immunity. We hypothesized that development of MCD involves an IL-33–related immune response. We examined 49 patients with biopsy-proven MCD, 6 healthy volunteers, and 29 patients in remission. In addition to clinical features, serum and urinary levels of IL-33 and soluble suppression of tumorigenicity 2 protein (sST2), a secreted form of the receptor of IL-33, were analyzed. Although IL-33 was barely detectable in either MCD or control samples, sST2 levels at diagnosis were elevated in MCD patients. Serum sST2 levels of MCD patients were correlated with serum total protein level (*r* =  − 0.36, *p* = 0.010) and serum creatinine level (*r* = 0.34, *p* = 0.016). Furthermore, the elevated sST2 levels were observed to decrease following remission. Immunofluorescence revealed IL-33 expression in the podocytes among MCD patients, with a significant increase compared with controls. In vitro, mouse podocyte cells incubated with serum from a MCD patient at disease onset showed increased IL-33 secretion. These results suggest an IL-33–related immune response plays a role in MCD.

## Introduction

Minimal change disease (MCD) is a major cause of idiopathic nephrotic syndrome (NS). MCD is reportedly responsible for 10–15% of idiopathic NS cases in adults and 70–90% of NS cases in children over 1 year of age^1^. Although corticosteroid treatment effectively induces remission in most cases, frequent relapse is a major clinical problem in patients with MCD. Although a number of studies have identified potential pathways or candidate molecules, the pathogenesis of proteinuria in MCD remains unclear^2^. Its frequent coexistence with bronchial asthma, atopic dermatitis, or a high titer of immunoglobulin (Ig) E suggests a possible clinical association between MCD and allergic conditions^3^ as well as type 2 cytokines such as interleukin (IL)-4, IL-5, and IL-13^4–6^.

Recent studies have revealed that cross-talk occurs between epithelial cells and immune cells. IL-33 is a member of the IL-1 cytokine family and reportedly plays a crucial role in type 2 innate immune responses^7,8^. IL-33 is expressed in the nucleus of epithelial cells and acts as an alarmin when released due to cell injury. It binds to its transmembrane receptor, suppression of tumorigenicity 2 (ST2), to stimulate ST2-positive immune cells, such as ILC2, Th2, and mast cells. Genome-wide association studies indicate that a polymorphism in the IL-33/ST2 gene is associated with bronchial asthma^9,10^. Indeed, IL-33 blockade led to a lower incidence of loss of asthma control and improved lung function^11^. The IL-33/ST2 axis also assume the important role within kidney milieu. It is reported that IL-33 induces ILC2 expression in mice^12,13^, which is the major subset of innate lymphoid cells in human and mouse kidney.

IL-33/ST2 signaling is negatively regulated by soluble ST2 (sST2), which lacks the transmembrane domain of full-length ST2 and functions as a decoy receptor for IL-33. Although the sources of circulating sST2 remain unknown^14^, endothelial cells^15^ or T cells^16^ are considered likely candidates. Some studies have reported that serum sST2 levels are increased in patients with bronchial asthma and correlated with disease severity^17–19^.

Despite recent advances in understanding of the relationship between IL-33 and type 2 immunity, the precise role of IL-33 in MCD remains unclear. This study focused on the behavior of the IL-33/ST2 axis in MCD and the possible role of IL-33 in the pathogenesis of proteinuria.

## Results

### IL-33 and sST2 concentrations at diagnosis among MCD patients

**Figure 1:**
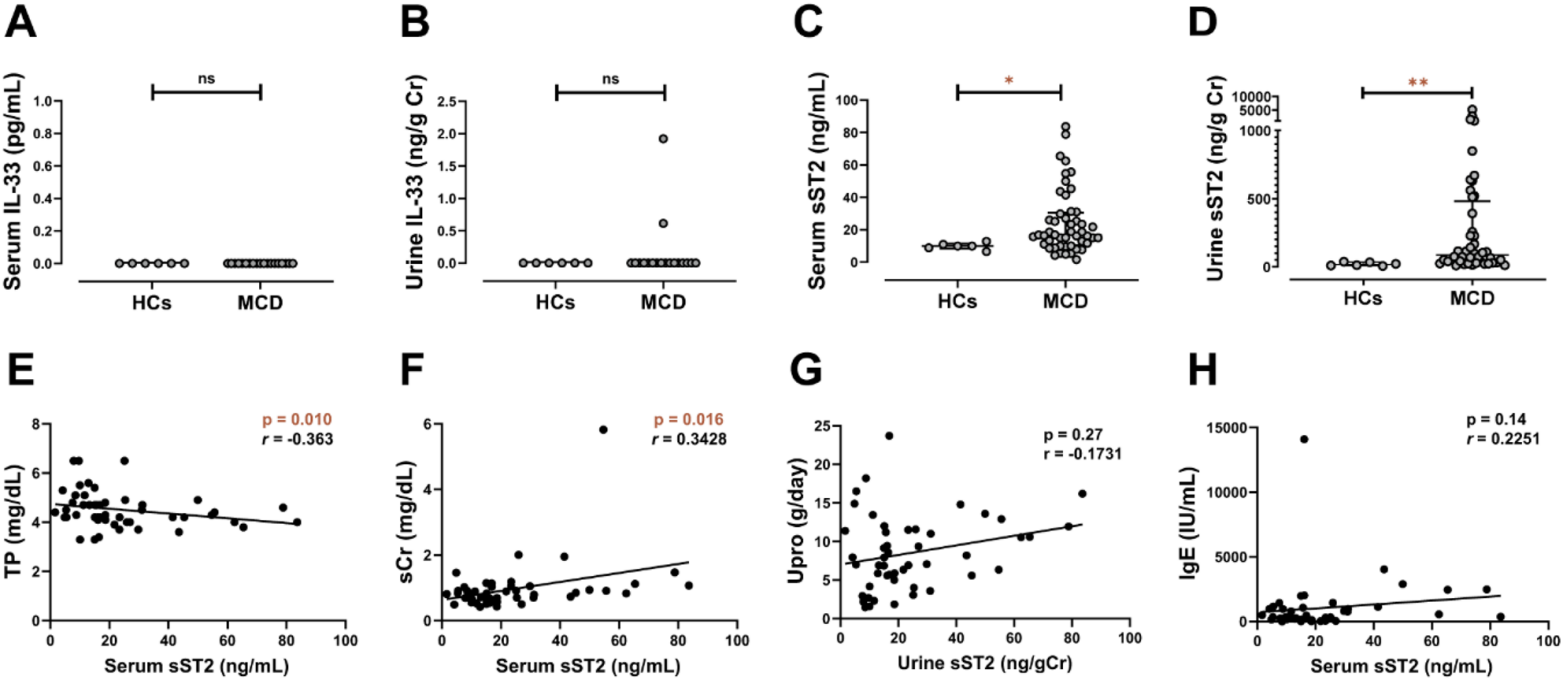
The sST2 levels were elevated in MCD at diagnosis and its serum levels were correlated with clinical features. (**A**, **B**) IL-33 was rarely detected in serum or urine samples of MCD patients (n = 19) or normal subjects (n = 6). (**C**) The serum sST2 level in MCD patients (n = 49) was significantly higher (16.9 ng/mL, IQR 10.6–30.4) than that of healthy controls (HCs) (n = 6) (9.94 ng/mL, IQR 8.27–11.4) (*p* = 0.017). (**D**) The urinary sST2 level in MCD patients (n = 44) was significantly higher (86.0 ng/g Cr, IQR 31.8–482) than that of HCs (n = 6) (16.3 ng/g Cr, IQR 8.32–34.1) (*p* = 0.0011). (**E**–**H**) The relationship between serum sST2 and various clinical features. Serum sST2 concentration was correlated with (**E**) serum total protein level (*p* = 0.010, Spearman’s *r* =  − 0.36) and (**F**) serum creatinine level (*p* = 0.016, Spearman’s *r* = 0.34). There were no significant correlations between levels of serum sST2 and (**G**) urinary protein, or (**H**) serum IgE. Horizontal lines from top down represent the 75th percentile, median, and 25th percentile. Abbreviations: *HCs*, healthy controls; *MCD*, minimal change disease; *TP*, total protein; *sCr*, serum creatinine; *Upro*, urinary protein excretion; *IgE*, immunoglobulin E. *ns*, not significant. **p < 0.01; *p < 0.05.

Serum and urinary levels of IL-33 were measured in 19 patients with MCD and 6 healthy volunteers, respectively (Fig. 1A,B). We rarely detected IL-33 in serum and urine samples from both MCD patients and normal subjects. On the other hand, the serum sST2 level at diagnosis among MCD patients (n = 49) was significantly higher (16.9 ng/mL, IQR 10.6–30.4) than that of healthy controls (n = 6) (9.94 ng/mL, IQR 8.27–11.4) (*p* = 0.017) (Fig. 1C). Urinary sST2 levels at diagnosis were also higher in MCD patients (n = 44) (86.0 ng/g Cr, IQR 31.8–482) than controls (n = 6) (16.3 ng/g Cr, IQR 8.32–34.1) (*p* = 0.0011) (Fig. 1D).

### Urinary IL-13 concentrations at diagnosis among MCD patients

We showed that the IgE levels of MCD patients were higher (n = 45) (368 IU/mL, IQR 161–1098) than reference value: 0–120 IU/mL (Table1). We measured the levels of urinary IL-13, which can induce isotype switching to IgE synthesis. However, no significant differences were observed in urinary IL-13 levels between HCs (n = 6) (9.25 ng/g Cr, IQR 0.00–44.1) and MCD patients (n = 44) (0.00 ng/g Cr, IQR 0.00–2.24) (*p* = 0.29).

**Table 1:** Characteristics of patients with MCD at the time of renal biopsy.

Variable	Total (n = 49)
Age (years)	43.6 (17.2)
Male, n (%)	23 (46.9)
Immunosuppresive therapy, n (%)	9 (18.4)
Systolic blood pressure (mmHg)	121 (18)
Diastolic blood pressure (mmHg)	76 (15)
Creatine (mg/dL)	0.97 (0.78)
eGFR (mL/min/1.73m^2^)	70.2 (25.5)
Urinary protein excretion (g/day)^a^	8.5 (4.9)
Total protein (mg/dL)	4.5 (0.7)
Albumine (mg/dL)^a^	1.53 (0.60)
Low-density lipoprotein cholesterol (mg/dL)	325 (122)
Hemoglobin A1c (%)^b^	5.5 (0.4)
Immunoglobulin E (IU/mL)^b^, median (IQR)	368 (161–1098)

Data are expressed as mean (SD), median (IQR), or number (%).

^a^Data were not available for 2 patients.

^b^Data were not available for 4 patients.

*eGFR*, estimated glomerular filtration rate.

### Correlation between sST2 concentrations and clinical parameters

The correlations between serum and urinary levels of sST2 and various clinical parameters associated with NS were also analyzed. Serum sST2 concentration was negatively correlated with the level of serum total proteins (Spearman’s *r* =  − 0.36, *p* = 0.010) (Fig. 1E) and positively correlated with the level of serum Cr (Spearman’s *r* = 0.34, *p* = 0.016) (Fig. 1F). However, there were no significant correlations between levels of serum sST2 and urinary protein (Fig. 1G) or serum IgE (Fig. 1H). No significant correlations were observed between urinary sST2 levels and the clinical parameters examined.

### Follow-up study of sST2

**Figure 2:**
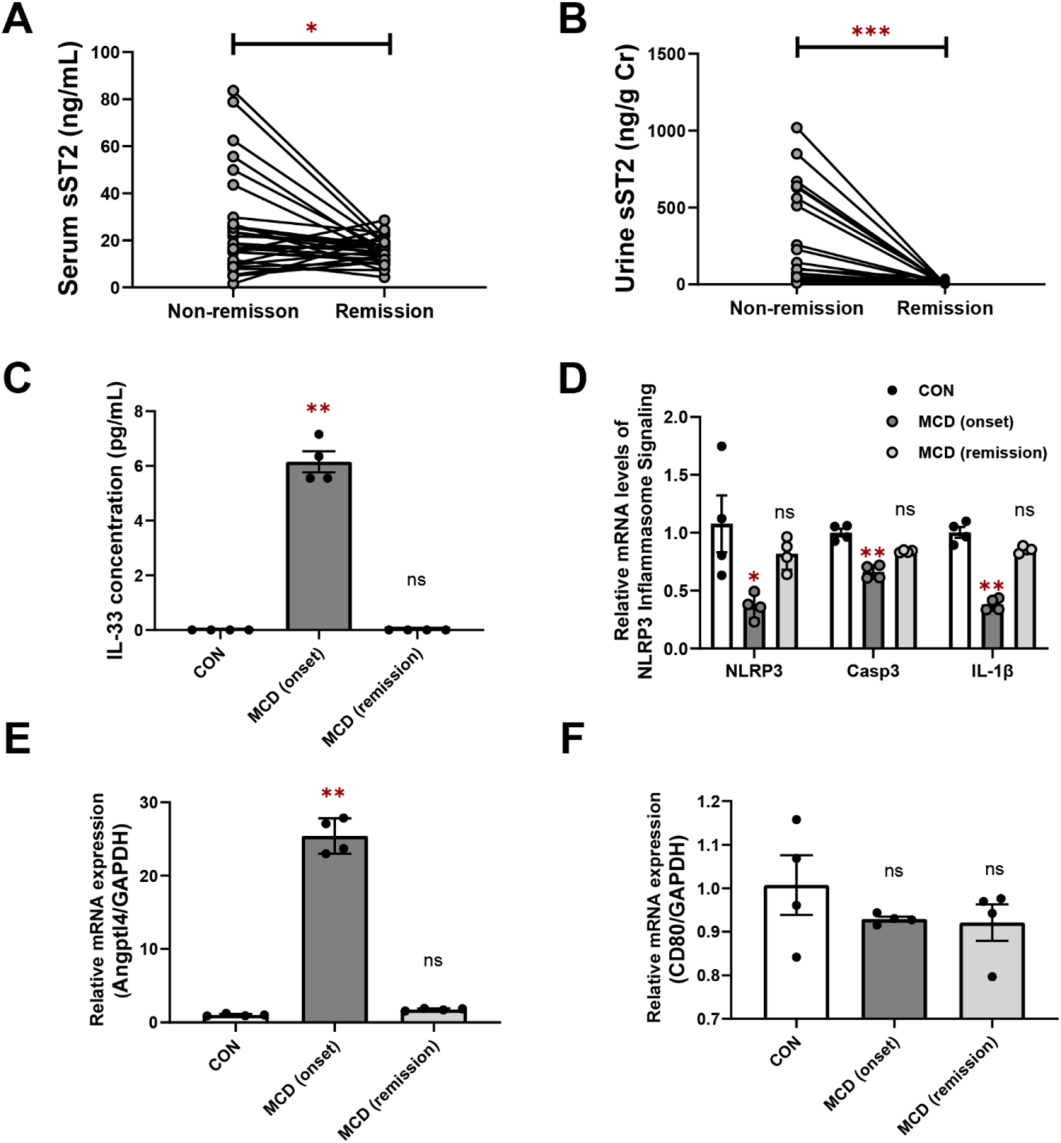
Follow-up study of sST2 levels in MCD patients and effects of MCD serum on cultured podocytes. (**A**) In 29 patients, a significant decrease in the serum sST2 level (ng/mL) was observed during remission (mean ± SEM = 25.6 ± 3.97 versus 15.6 ± 1.02; median 18.6 versus 15.4; Wilcoxon *p* = 0.018). (**B**) In 26 patients, a significant decrease in the urinary sST2 level (ng/g Cr) was observed during remission (mean ± SEM = 247 ± 61.1 versus 5.48 ± 1.72; median 72.4 versus 2.18; Wilcoxon *p* < 0.0001). (**C**–**F**) Differentiated conditional immortalized mouse podocyte cells were stimulated for 6 h with 15% serum obtained from a patient with MCD. (**C**) IL-33 concentration in the podocyte culture supernatant. Real-time RT-PCR analysis of (**D**) NLRP inflammasome signaling, (**E**) Angptl4 and (**F**) CD80. Data are expressed as mean ± SEM. ***p < 0.001; **p < 0.01; *p < 0.05.

All patients diagnosed with MCD received immunosuppressive therapy with corticosteroids, cyclosporin, and/or rituximab. We evaluated sST2 concentrations in serum (n = 29) and urine (n = 26) samples during remission. Patients with MCD showed a significant reduction in sST2 levels in the serum (mean ± SEM = 25.6 ± 3.97 versus 15.6 ± 1.02; median 18.6 versus 15.4; *p* = 0.018) and urine (mean ± SEM = 247 ± 61.1 versus 5.48 ± 1.72; median 72.4 versus 2.18; *p* < 0.0001) during remission (Fig. 2A,B).

### Localization of IL-33 expression in glomeruli

**Figure 3:**
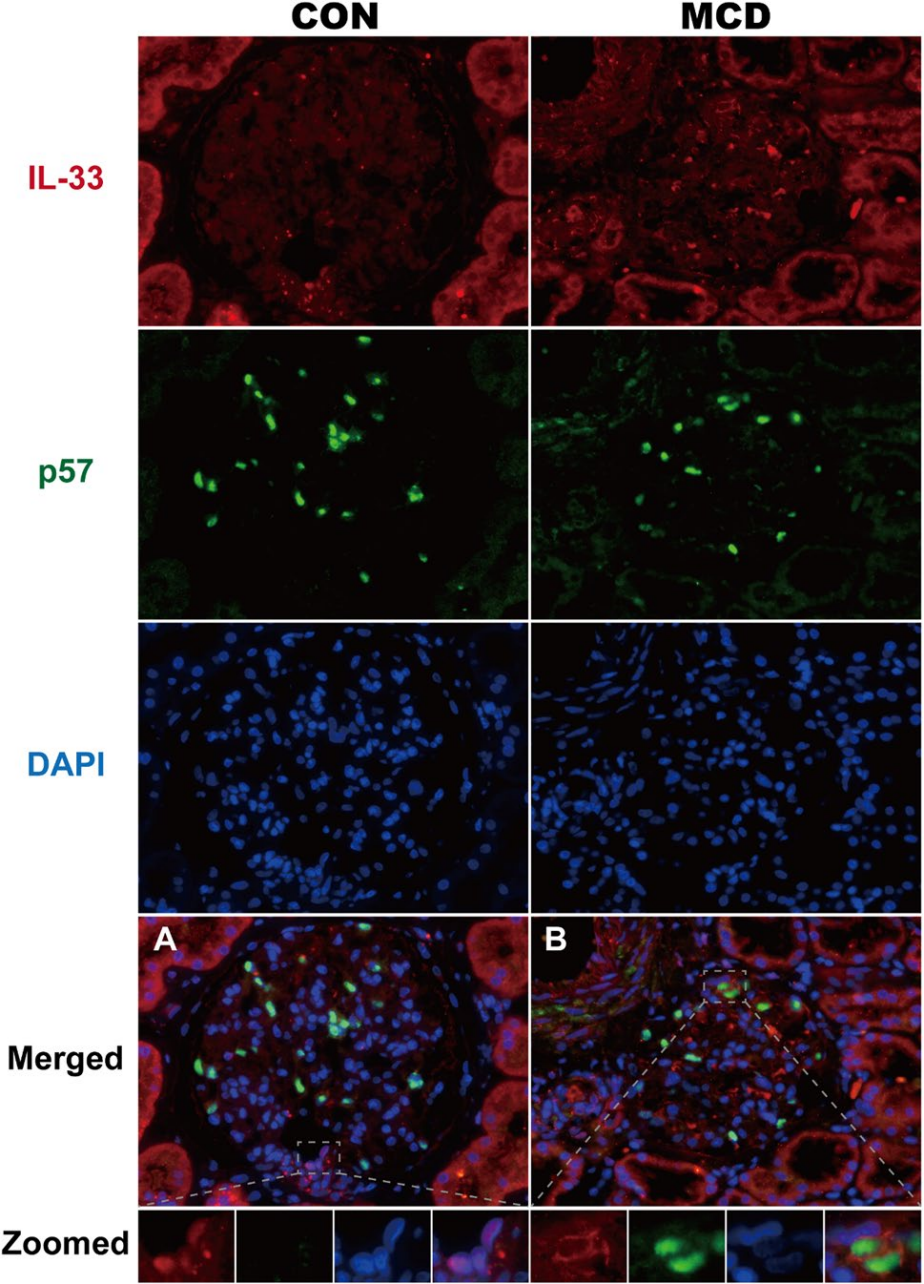
Localization of IL-33 and p57 in glomeruli. Representative images of kidney biopsy specimens of control kidney (CON) and MCD tissue stained for IL-33 (red) and p57 (green) and counterstained with DAPI (blue). (**A**) IL-33 was observed in the nucleus of endothelial cells of glomerular vascular poles, as well as arteries and arterioles (not shown). (**B**) In patients with MCD, IL-33 was detected in the cytoplasm of glomerular epithelial cells confirmed as positive for the podocyte nuclear marker p57. Original magnification × 400.

We hypothesized that the IL-33/ST2 axis plays a role in kidney tissues in MCD, similar to that in the endobronchial tissue of asthma patients^20^. To determine the expression profile and potential cellular source of IL-33 in kidney tissue, we examined IL-33 protein expression in glomeruli using immunofluorescence analysis of sections of renal biopsy specimens. IL-33 was observed in the nucleus of endothelial cells among arteries or arterioles, including glomerular vascular poles (Fig. 3A). IL-33 expression was also detected in the cytoplasm of glomerular epithelial cells identified by positive staining for the podocyte nuclear marker^21^ p57 (Fig. 3B). The number of cells double-positive for p57 and IL-33 per glomerular cross-section was significantly higher in MCD patients than controls (mean ± SEM = 0.345 ± 0.169 versus 0.065 ± 0.025; *p* = 0.027).

### IL-33 expression in cultured glomerular epithelial cells stimulated with serum from an MCD patient

We speculated that some substance in the serum of MCD patients stimulates the secretion of IL-33 by glomerular epithelial cells. To consider the possible roles of IL-33 in glomerular epithelial cells, we investigated whether IL-33 expression was increased in cultured MPCs stimulated with serum obtained from a patient with MCD. Stimulation for 6 h with serum obtained at disease onset increased the secretion of IL-33 into the culture supernatant (*p* = 0.0100; Fig. 2C), whereas there was no significant increase in IL-33 secretion by cells stimulated with serum from a patient in remission. To comprehensive the regulatory mechanism underlying IL-33, we evaluated the gene expression of NLR family pyrin domain containing 3 (NLRP3) inflammasome, along with its subsequent signaling cascade. Treatment of podocytes with serum from a patient did not stimulate NLRP3 inflammasome signaling and resulted in the inverse effects on NLRP3, Casp1, or IL-1β gene expression levels (*p* = 0.022, *p* = 0.0034, and *p* = 0.015 respectively; Fig. 2D). We also examined expression of the genes encoding Angptl4 and CD80, which are considered mediators of proteinuria in MCD. Stimulation with serum obtained at disease onset increased Angptl4 gene expression (*p* = 0.0034; Fig. 2E). In contrast, there was no significant increase in CD80 gene expression (Fig. 2F).

## Discussion

Although recent studies have revealed the mechanism of IL-33–related immune responses and type 2 immunity in allergic conditions, the role and function of IL-33 and ST2 in kidney diseases is poorly understood. We demonstrated that sST2 levels are elevated in patients with MCD and that serum sST2 levels are correlated with disease severity and activity of NS. Furthermore, IL-33 expression was detected on the surface of glomerular epithelial cells in kidney biopsy specimens, and upregulation of IL-33 was observed in cultured MPCs stimulated with serum obtained from an MCD patient. To the best of our knowledge, this is the first report to describe the potential role of the IL-33/ST2 axis in proteinuria of MCD.

IL-33 is considered an important modulator of immune responses against invading pathogens or other environmental insults due to its predominant distribution in the epithelial barrier^22^. IL-33 released from damaged or necrotic cells acts as an alarmin that stimulates ST2-positive cells. Various studies have demonstrated the role of IL-33 in innate allergic airway responses using ST2- or IL-33–deficient mice and identified lung ILC2 as the primary target of IL-33 in the airway to induce a type 2 immune response. Clinical studies have reported elevated IL-33 protein levels in sputum^19^, bronchoalveolar lavage fluids, and serum^19^ of bronchial asthma patients, with IL-33 levels generally correlating with disease severity^22^. We hypothesized that IL-33 plays a role in the proteinuria of MCD coexisting with an allergic condition. Counter to our expectation, IL-33 was rarely detected in MCD patients or healthy controls in this study. However, whether serum or urine IL-33 levels are elevated in patients with MCD should be carefully determined, as elevated levels of soluble ST2 in human serum have been shown to interfere with IL-33 quantification by enzyme-linked immunosorbent assay (ELISA)^23^, and the sensitivity and specificity of available ELISA kits are not sufficient for evaluating serum IL-33 levels^24^. In addition, the potential effect of posttranslational modifications through cathepsins or elastase on IL-33 detection should also be considered^25^. Generally, discerning cytokines circulating at very low levels in blood is not consistently straightforward^26^. Regarding IL-13, a prototypical Type 2 cytokine, its circulating concentrations are not consistently high within the context of allergic conditions^26,27^. It is proposed that evaluating IL-13 concentrations locally at the diseased anatomical site holds greater relevance^26^. In this study, we measured urinary IL-13 levels in MCD patients, however, no significant differences were observed between HCs and MCD patients. Further study is needed to comprehensively assess their status.

Some studies have reported that levels of serum sST2, the soluble form of the IL-33 decoy receptor, are elevated in patients with asthma and correlated with disease severity^17–19^. In this study, we demonstrated that sST2 levels were significantly elevated in serum and urine samples obtained from patients with MCD. Serum sST2 levels were positively correlated with serum total protein levels and negatively correlated with serum creatinine levels. Although we could not observe a correlation between sST2 levels and proteinuria directly, we speculated that serum sST2 levels are correlated with disease severity in NS. Furthermore, we found that serum and urine levels of sST2 were decreased after the onset of remission, corresponding with disease activity in patients with MCD. We speculated that sST2 expression is upregulated in IL-33–related immune responses as a form of negative feedback, and these results might therefore reflect the severity of the type 2 immune response. Type 2 immunity induces a complex inflammatory response that could become an important disease driver.

Préfontaine et al. reported that IL-33 expression by epithelial cells is increased in bronchial asthma^20^. In this study, we observed that IL-33 was expressed on glomerular epithelial cells confirmed as positive for expression of the podocyte nuclear marker p57^21^. IL-33 expression on glomerular epithelial cells was significantly increased among patients with MCD, suggesting that IL-33 plays a role in the blood-urine barrier in glomerular epithelial cells. IL-33 is a nuclear protein lacking a signal sequence, unlike conventional cytokines, and it functions as a rapid-acting alarmin that activates immune cells. Although excessive necrotic change is not thought to occur in glomerular epithelial cells in MCD, some studies have revealed that IL-33 can be released from the cytoplasm without cell death by increasing the intracellular calcium concentration through ATP administration^28^. Interestingly, we found that IL-33 expression was localized predominantly in the cytoplasm of glomerular epithelial cells rather than the nucleus. Gordon et al. demonstrated that IL-33 alternative splice variants localized to different cellular compartments in primary human airway epithelial cells^29^; that is, full-length IL-33 localized to the cell nucleus, whereas variants resulting from deletion of exons 3 and 4 localized to the nucleus and cytoplasm. They showed that ∆exon 3, 4 variants are secreted by ionomycin without cell death to a greater extent than the full-length protein and that this is strongly associated with airway expression of type 2 cytokine genes in asthma. Although the distribution of IL-33 was not identical to that in airway epithelial cells in terms of negativity in the nucleus, we speculate that IL-33 might be secreted from the cytoplasm of glomerular epithelial cells in the absence of cell death in MCD.

**Figure 4:**
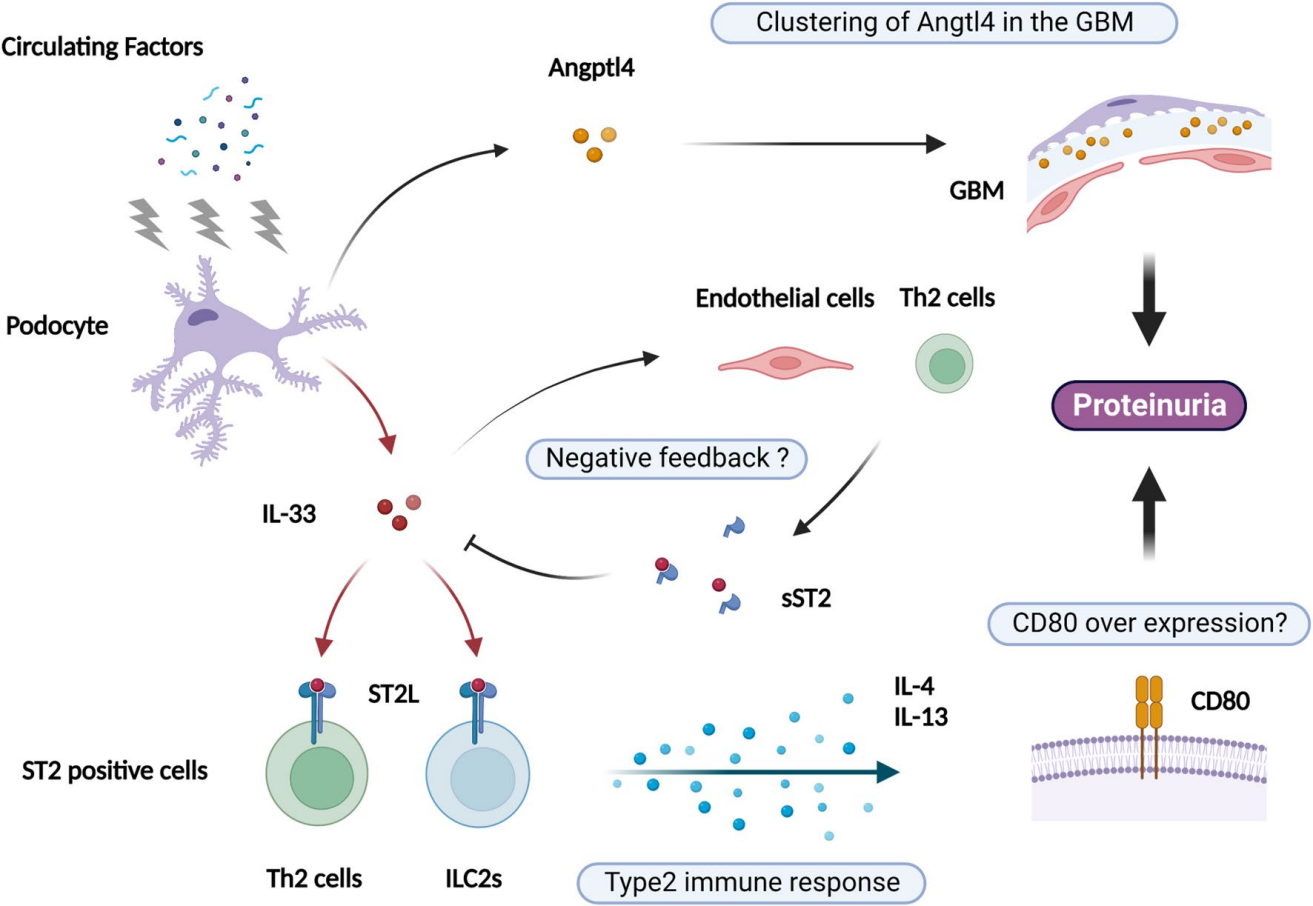
Schematic illustration of the IL-33 immune response in MCD. Circulating factors target podocytes, resulting in secretion of IL-33 and Angptl4. IL-33 stimulates ST2-positive immune cells to induce a type 2 immune response. Type 2 cytokines such as IL-13 might induce overexpression of CD80, which is considered a mediator of proteinuria in MCD. In contrast, Angptl4 secreted by podocytes reportedly clusters in the GBM, resulting in effacement of foot processes and acceleration of proteinuria. Abbreviations: *IL-33*, interleukin-33; *sST2*, soluble suppression of tumorigenicity 2 protein; *ST2L*, suppression of tumorigenicity 2 protein ligand; *ILC2*, group 2 innate lymphoid cells; *Th2 cells*, T helper 2 cells; *IL-4*, interleukin-4; *IL-13*, interleukin-13; CD80, cluster of differentiation 80; Angptl4, angiopoietin-like protein 4; GBM, glomerular basement membrane. Created using BioRender.com.

We also found that treatment of cultured mouse glomerular epithelial cells with serum obtained from a patient at the onset of MCD led to an increase in the IL-33 concentration in the supernatant. This result suggests that some substance in the serum stimulates the secretion of IL-33 by podocytes in MCD and confirms our hypothesis that IL-33 plays a role in the blood-urine barrier in glomerular epithelial cells. Multiple stressors can induce podocytes to acquire features of immune cells^30,31^. CD80 is a transmembrane protein normally expressed on antigen-presenting cells to regulate the immune response^30^. Expression of CD80 is reportedly also upregulated in podocytes of rodent NS models, thereby contributing to the pathogenesis of proteinuria^32^. Lai et al. reported that, in Wistar rats, overexpression of IL-13, a type 2 cytokine induced by IL-33, results in minimal change–like nephropathy, which is characterized by severe proteinuria and fusion of podocyte foot processes with upregulation of CD80 expression in glomeruli^33^. Furthermore, Garin et al. showed that CD80 levels are elevated in urine from MCD patients^34^; the source of this elevated CD80 could be podocytes^35^. Although the significance of CD80 in MCD remains controversial^30^, elucidating the function of this molecule could help explain the role of type 2 cytokines in MCD. In this study, we did not observe an upregulation of CD80 mRNA expression in cultured podocytes stimulated with serum from an MCD patient at disease onset, as opposed to Angptl4, another candidate mediator of MCD^2,36^. We speculated that IL-33 secreted by podocytes induces the activation of type 2 cytokines produced by ILC2 or Th2 cells to induce proteinuria (Fig. 4).

Recently, it has been sighted that the inflammasome component of NLRP3 regulate inflammation, pyroptosis, apoptosis, and fibrosis in kidney diseases^37^. R Fu et al. reported that NLRP3 inflammasome was activated in podocytes from lupus-prone mice and patients with LN^38^. Although there is not strong evidence for an interaction of the NLRP3 inflammasome and IL-33/ST2 axis^39^, some studies have proposed the NLRP3/IL-33/ST2 axis^40,41^. Ma et al. reported the positive correlation between the IL-33 and NLRP3 in patient with lupus nephritis^41^. In this study, we did not observe that treatment of podocytes with serum from a MCD patient enhanced NLRP3 inflammasome signaling. Further studies will be needed to fathom the association between NLRP3 and IL-33/ST2.

There are some limitations in this study. Firstly, because the allergic patients without MCD were not enrolled as controls, it is difficult to determine how much effect MCD without allergic conditions has on the levels of sST2. Secondly, due to the quantification of IL-33 levels utilizing a commercially procurable ELISA kit, the potential impact stemming from elevated sST2 concentrations within the serum or urine samples could not be mitigated. IL-33 biding with sST2 poses heightened complexity in its discernibility through the utilization of ELISA. Thirdly, our results of cell culture study were based on murine podocyte cell line. The more optimal approach for assessing the reaction of glomerular epithelial cells to human serum should be based on human podocyte. Finally, we could not definitively exclude the potential influence of complement factors on our in vitro findings, as we exposed human serum to experimentation without prior inactivation.

In conclusion, we demonstrated for the first time that levels of sST2, the soluble form of the IL-33 receptor, are elevated in the serum and urine of patients with MCD. IL-33 was detected in glomerular epithelial cells of MCD patients, and cell culture experiments revealed that serum obtained from an MCD patient could stimulate IL-33 secretion by murine glomerular podocytes. Our results suggest a potential role for the IL-33/ST2 axis in the development of MCD and should enhance understanding of the mechanism underlying proteinuria in MCD in terms of type 2 immune responses.

## Methods

### Patients and samples

All patients and their legal guardians provided written informed consent regarding preservation of blood samples, urine samples, and kidney biopsy specimens. Opt-out methods were used to obtain informed consent regarding the measurement of IL-33/sST2 values at diagnosis and evaluation of IL-33 deposition. All enrolled patients agreed to participate in this study. The protocol of this study was established according to the Declaration of Helsinki and approved by the Showa University Research Ethics Review Board (No. 3118). A total of 49 patients with MCD admitted to Showa University Hospital (Tokyo, Japan) between April 2009 and March 2022 were selected for the study (Table 1). The diagnosis of MCD was based on renal biopsy findings. All patients had proteinuria > 1.0 g/day. Blood samples were collected from all patients at the time of renal biopsy, and urine samples were obtained from 44 patients. Blood samples were collected from 29 patients in complete remission, and urine samples were obtained from 26 of these patients.

### Enzyme-linked immunosorbent assay

The serum and urine concentrations of IL-13, IL-33 and sST2 were measured using commercially available ELISA kits (R&D Systems, San Diego, CA, USA), according to the manufacturer’s instructions. All urine measurements were normalized to urinary creatinine (Cr) levels.

### Immunofluorescence staining

We selected kidney biopsy specimens diagnosed as MCD (n = 7) with a sufficient number of glomeruli (> 5) and 0-h protocol biopsy specimens of kidney grafts (n = 7) without glomerular lesions, and the latter were used as normal controls. Formalin-fixed, paraffin-embedded kidney biopsy specimens were sectioned (3-µm thick), deparaffinized, and washed in phosphate buffered saline (PBS). Antigen-retrieval solution (0.01 mol/L citrate buffer; pH 6.0) was heated in a microwave oven at 500 W of power for 5 min, and then the tissue sections were placed in the solution and heated in the microwave oven at 500 W of power for 9 min. After cooling at room temperature for 30 min, the sections were washed three times with PBS for 2 min each. Background Buster^®^ (Innovex Biosciences, Richmond, CA, USA) was applied to the sections and incubated at room temperature for 15 min to block nonspecific background staining. The sections were then washed three times with PBS and incubated with goat polyclonal anti-human IL-33 antibody (working dilution 1:100; R&D Systems) at 4 °C overnight. After washing with PBS, the sections were incubated with donkey polyclonal anti-goat IgG H&L (Alexa Fluor^®^ 647) (working dilution 1:200; Abcam, Cambridge, UK) for 60 min at room temperature. The sections were then washed with PBS and incubated with mouse monoclonal anti-p57 Kip2 antibody (working dilution 1:600; Santa Cruz Biotechnology, Santa Cruz, CA, USA) at 4 °C overnight. After washing, the sections were incubated with goat polyclonal anti-mouse IgG (H + L) (Alexa Fluor^®^ 488) (working dilution 1:200; Thermo Fisher Scientific, Waltham, MA, USA) at room temperature for 60 min. Finally, the sections were counterstained with 4′6-diamidino-2phenylindole (DAPI) (Vector Laboratories, Newark, CA, USA). The nuclei of arteriole or small artery endothelial cells, which typically stain positive, were used as internal controls for IL-33 staining^42^.

### Quantification of immunostaining

Image acquisition was performed using a BZ-X800 all-in-one fluorescence microscope (Keyence, Osaka, Japan). All images were processed using ImageJ software, version 1.53a. Podocyte cells were identified based on p57 staining of nuclei^21,43^. The number of podocytes double-positive for IL-33 and p57 per glomerular cross-section was also determined. An average of 39.7 control glomeruli and 16.3 MCD glomeruli were assessed in this study.

### Cell culture study

Conditionally immortalized mouse podocyte cells (MPCs) were kindly donated by Dr. Peter Mundel (Boston, MA, USA). MPCs were grown in RPMI-1640 (Thermo Fisher Scientific) containing 10% fetal bovine serum and 1% streptomycin-penicillin mixture (Thermo Fisher Scientific) in an atmosphere 5% CO_2_ and 95% air. MPCs were cultured for propagation at 33 °C with 10 U/mL of recombinant mouse γ-interferon (Merck, Darmstadt, Germany). Upon reaching 80% confluence, the MPCs were shifted to 37 °C without γ-interferon for 10 days to induce differentiation. Differentiated MPCs were stimulated for 6 h with 15% serum from a patient with MCD (Supplemental Table 1). Serum was directly added to the MPCs without activation of complements^44^. Expression of the mouse angiopoietin-like protein 4 (Angptl4), cluster of differentiation (CD)80, NLR family pyrin domain containing 3 (NLRP3), Caspase 1 (Casp1), and GAPDH genes were analyzed using real-time reverse transcription–polymerase chain reaction as previously described^45^. Levels of mouse IL-33 in the supernatant were determined using an ELISA kit (R&D Systems), according to the manufacturer's instructions.

### Statistical analysis

Quantitative data are expressed as mean and standard deviation (SD), median and interquartile range (IQR), or mean ± standard error of the mean (SEM), as appropriate. Qualitative data are presented as absolute numbers and percentages. Differences between the groups were evaluated using the Mann–Whitney *U* test or Kruskal–Wallis test. Correlations between various parameters were analyzed using the Spearman’s rank correlation test. Differences in sST2 levels between the time of diagnosis and onset of remission were analyzed using the Wilcoxon signed rank test. All statistical analyses were performed using GraphPad Prism software, version 8 (GraphPad Software, La Jolla, CA, USA), and values of *p* < 0.05 were considered statistically significant.

### Supplementary Information

Supplementary Table 1.

## Data Availability

The data presented in this study are available from corresponding author on reasonable request.
