# Publisher Correction: Exploring the significance of interleukin-33/ST2 axis in minimal change disease

**DOI:** 10.1038/s41598-023-47970-4

**Published:** 2023-11-24

**Authors:** Nobuhiro Kanazawa, Masayuki Iyoda, Taihei Suzuki, Shohei Tachibana, Ryuichi Nagashima, Hirokazu Honda

**Affiliations:** 1 Division of Nephrology, Department of Medicine, Showa University School of Medicine, Tokyo, Japan; 2 Department of Microbiology and Immunology, Showa University School of Medicine, 1-5-8 Hatanodai, Shinagawa-ku, Tokyo, 142-8555 Japan

Correction to: *Scientific Reports* 10.1038/s41598-023-45678-z, published online 31 October 2023

The original version of this Article contained an error in the Discussion section, where a part of the Legend of Figure 4 was incorrectly duplicated in the main body.

The original Article has been corrected.

