# Supplementary material for: Exploring the significance of interleukin-33/ST2 axis in minimal change disease
Source: Sci Rep. 2023 Oct 31;13:18776. doi: 10.1038/s41598-023-45678-z (PMC10618262; doi:10.1038/s41598-023-45678-z)
Supplement: Supplementary file 1 — Supplementary Table 1. [file 41598_2023_45678_MOESM1_ESM.pdf]

Exploring the significance of interleukin-33/ST2 axis in minimal change disease

Nobuhiro Kanazawa[1], Masayuki Iyoda[1,2*], Taihei Suzuki[1], Shohei Tachibana[1], Ryuichi Nagashima[2], and Hirokazu Honda[1]

[1] Division of Nephrology, Department of Medicine, Showa University School of Medicine, Tokyo, Japan

[2] Department of Microbiology and Immunology, Showa University School of Medicine, Tokyo, Japan

Variable	Patient
Gender (Male/Female)	Female
Age (year)	26
SBP (mmHg)	88
DBP (mmHg)	65
Cr (mg/dl)	0.58
eGFR (ml/min/1.73m2)	102.1
Upro (g/day)	5.89
TP (mg/dl)	4.1
Alb (mg/dl)	1.4
LDL-C (mg/dl)	216
HbA1c (%)	5.2
WBC (10*3/μl)	5300
Neut (10*3/μl)	3150
Lym (10*3/μl)	1700
Mono (10*3/μl)	159
Eo (10*3/μl))	133
IgE (IU/mL)	117

Supplemental Table

Clinical information of a patient who donated serum samples.

Abbreviation: *SBP*, systolic blood pressure; *DBP,* diastolic blood pressure; *Cr*, creatinine; *eGFR*, estimated glomerular filtration rate; *Upro*, urinary protein excretion; *TP*, total protein; *Alb*, albumin; *LDL-C*, low-density lipoprotein cholesterol; *HbA1c*, hemoglobin A1c; *WBC*, white blood cell; *Neut*, neutrophil; *Lym*, lymphocyte; *Mono*, monocyte; *Eo*, eosinophil; *IgE*, immunoglobulin E.
